# SARS-CoV-2 infections in infants in Haiti 2020–2021; evidence from a seroepidemiological cohort

**DOI:** 10.1371/journal.pone.0273482

**Published:** 2022-08-25

**Authors:** Rigan Louis, Ruiyu Pu, Tracey D. Logan, Luke Trimmer-Smith, Richard Chamblain, Adriana Gallagher, Valery Madsen Beau De Rochars, Eric Nelson, Derek A. T. Cummings, Maureen T. Long, J. Glenn Morris

**Affiliations:** 1 Emerging Pathogens Institute, University of Florida, Gainesville, FL, United States of America; 2 Faculte de Medicine et de Pharmacie, Universite d’Etat d’Haiti, Port-au-Prince, Haiti; 3 Department of Comparative Diagnostic and Population Medicine, College of Veterinary Medicine, University of Florida, Gainesville, FL, United States of America; 4 Department of Health Services Research, Management and Policy, College of Public Health and Health Professions, University of Florida, Gainesville, FL, United States of America; 5 Department of Pediatrics, College of Medicine, University of Florida, Gainesville, FL, United States of America; 6 Department of Environmental and Global Health, University of Florida, Gainesville, FL, United States of America; 7 Department of Biology, College of Liberal Arts and Sciences, University of Florida, Gainesville, FL, United States of America; 8 Department of Medicine, College of Medicine, University of Florida, Gainesville, FL, United States of America; University of Cape Town, SOUTH AFRICA

## Abstract

Few data are available on frequency of SARS-CoV-2 infection among very young children in low- to middle-income countries (LMIC), with the studies that are available biased towards higher income countries with low reported infection and seroconversion rates. Between February 2019 and March 2021, 388 dried blood spot (DBS) samples were obtained from 257 children less than 30 months of age as part of a prospective observational cohort study of pregnant women and their infants in Haiti; longitudinal samples were available for 107 children. In a subsequent retrospective analysis, DBS samples were tested by ELISA for antibody targeting the receptor binding domain of the SARS-CoV-2 S1 protein. Over the course of the study, 16·7% of the infants became seropositive. All seropositive samples were collected after March 19, 2020 (the date of the first reported COVID-19 case in Haiti) with the highest hazards measured in August 2020. Sampling date was the only covariate associated with the hazard of seroconversion. Our data provide an estimate of SARS-CoV-2 infection rates among very young children without prior SARS-CoV-2 exposure during the initial pandemic waves in Haiti, and demonstrate that these children mount a detectable serological response which is independent of patient age.

## Introduction

While there have been a number of population-based serologic studies that have assessed rates of SARS-CoV-2 infection in the developed world, available seroprevalence data are more limited in the developing world [1–4]. Of the 968 studies included in a recent meta-analysis, the majority (77%) of papers retained for analysis were from high income countries, with high income countries having a lower seroprevalence than low to middle income countries (LMIC) [1]. Most of the papers that have been published, irrespective of income, have focused on adult populations, with limited published data on infections among the youngest age groups [3–6]. In the limited studies available with age stratification under the age of two years, children were generally reported as seronegative [7, 8]. In a study conducted in Lima, Peru children ages 5–9 had a 20·3% seropositivity rate which was similar to other age groups including working and aged participants, suggesting that children, at least in this age range, have the same degree of exposure to SARS-CoV-2 as do adults in LMIC settings [5]. Infected infants less than two years of age have been reported to have SARS-CoV-2 viral loads compared to those seen in older children and adults [9]. In this setting there is a need to understand the rate of infection and immune responses amongst infants to assist in development of appropriate surveillance, intervention strategies and disease control models.

**Fig 1:**
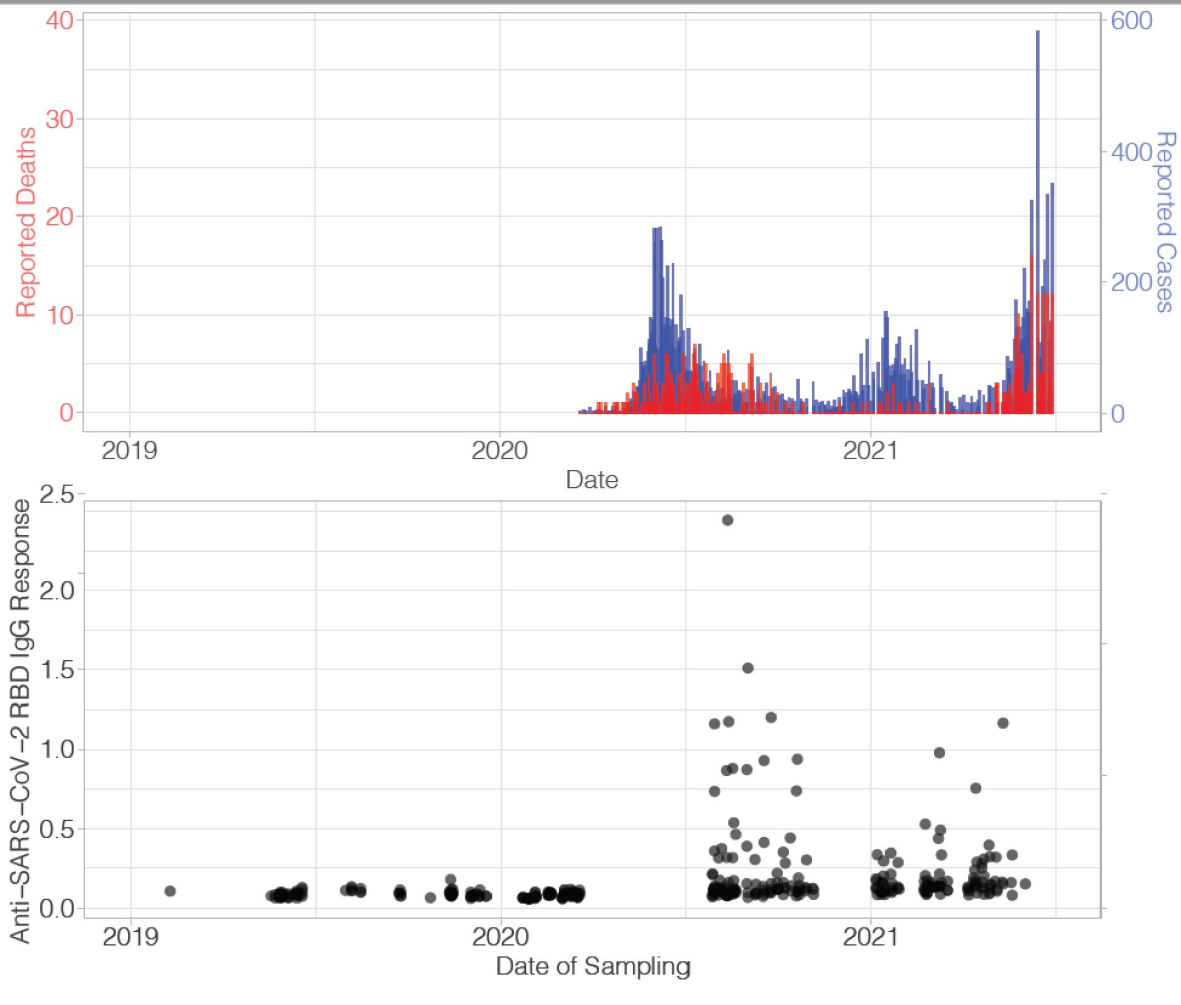
Reported cases (blue) and deaths (red) due to SARS-CoV-2 in Haiti (top) and optical density from ELISA that measures the antibody to SARS-CoV-2 Receptor Binding Domain IgG (bottom) by date of sampling for Haitian infants born between March 2019 and May 2021 and followed for up to 30 mos.

The first case of COVID-19 in Haiti was identified on March 19, 2020. As part of an international multi-site study to assess the impact of arboviral infection on pregnancy outcome (the ZIKAction project) [10], our group monitored a maternal and child cohort in the Gressier region of Haiti beginning in April, 2018. Infants of enrolled mothers were followed subsequently for up to 30 months of age, with efforts made to collect dried blood spot (DBS) samples at each visit; samples were collected from February 2019 through March 2021. Serendipitously, this time period included the first reported Haitian COVID-19 cases and the peaks of the first and second Haitian COVID-19 “waves” (Fig 1). We used these data and samples to assess serological responses to SARS-CoV-2 among infants pre-pandemic and during the pandemic in 2020 and 2021 in Haiti. In parallel work conducted and reported by our group [11] the predominant SARS-CoV-2 lineage circulating in Haiti during the study was B.1, with smaller numbers of cases in the B1.478 and B1.1 lineages. COVID-19 vaccines were not available for either mothers or infants in Haiti during the time period of the study.

## Methods

### Study design

This study was conducted under review of the University of Florida (UF) Institutional Review Board #201601229 and the Comite National de Bioethique of the Ministere De La Sante Publique Et De La Population. Sampling and handling of sera were conducted under the guidelines and review of UF Environmental Health and Safety. Signed informed consent was provided by all participants.

Mothers in the study were recruited from an antenatal clinic in Gressier, Haiti, between April 2018 and August 2019. Inclusion criteria included age of 18 years or older, pregnancy, the ability to provide informed consent, and no known medical or psychological problems. Basic demographic and epidemiologic data were obtained from mothers at the time of enrollment: maternal variables included age, parity, marital status, level of education, household demographics, district of sample collection, and type of setting (urban vs. rural). Efforts were made to collect serum samples from mothers at the time of enrollment, at 28 weeks, and at delivery; the last maternal serum sample was collected December 27, 2019, before the first reported Haitian COVID-19 case in March, 2020. Approximately two-thirds of the women approached about joining the study agreed to participate; 542 women were enrolled, with 308 (57%) completing the study with a live birth.

Efforts were made to follow infants born to mothers enrolled in the study, with visits scheduled at 0, 4, 12, and 18–30 months of age. At the time of visits, infants were screened for possible arbovirus-related congenital abnormalities and developmental delays, and DBS samples (FTA Micro DBS card, GE Healthcare–Whatman, Fisher Scientific) were collected by heel-stick. Mothers were asked to report illness in their infants since the infant was last seen by study staff. Travel within Haiti was severely limited during the study period because of substantial political unrest and, for the latter part of the study period, an active COVID epidemic. Consequently, follow-up of infants was not optimal: mothers often elected to deliver at home rather than attempt to reach a hospital or birthing center, and follow-up visits for infants were frequently missed. Many mothers were also hesitant about allowing a heel-stick for blood sample collection for DBS.

### Elution of DBS samples

Laboratory studies were conducted at the Emerging Pathogens Institute, University of Florida. From the DBS card, a single 6 mm biopsy punch was used to capture the area covered with each blood spot. Each punch was added to a 1·0 ml microcentrifuge tube and submerged in 100 μl PBS-0·05% Tween per punch and incubated with rocking at 50 rpm at 4°C overnight. After incubation, the tubes were centrifuged at 10,500 X g for 2 minutes and after incubation the supernatant removed from the paper, transferred in a new microcentrifuge tube and frozen at -80°C until use.

### Protein quantification

Because the blood spots varied on each card in terms of size and intensity, we determined the amount of total protein in each sample. The protein concentration was measured using a Bradford protein microassay in all DBS samples obtained in 2019 and 2020. A protein standard set consisting of BSA diluted to a working range of 0·25 to 2·0 μg/ml were created. Three two-fold dilutions of each sample were made by diluting each in PBS starting at 1:5. A commercial Bradford reagent (Maker) was added as a 1:1 dilution to each sample and incubated for 5 minutes. Absorbance was measured at 595 nm. The final protein concentration was determined by creating a standard curve plotting the 595 nm value of each BSA dilution by its corresponding concentration. The unknown concentration of each sample was calculated from the curve as well as multiplying by the dilution factor.

### SARS-CoV-2 testing

A research ELISA targeting the Receptor Binding Domain (RBD) of the virus was used to detect exposure to SARS-CoV-2. The ELISA was adapted from a previously published protocol, which targets the RBD of the spike protein [12]. Briefly, 96-well ELISA plates were coated with 1 μg/ml RBD protein diluted in carbonate/bicarbonate buffer (pH 9·6) and incubated at room temperature (RT) for 1 hour. Each plate was then blocked with 1X Tris-buffered saline (TBS) with 5% milk and incubated for 2 hours. After blocking, each sample, diluted at a concentration of 1:100 in TBS-0·5% Tween, was added in duplicate to the plate. Mouse anti-human IgG-HRP (Jackson Immunoresearch, 109-035-098) was then added and incubated for 1 hour. After incubation and washing, 3,3’,5,5’tretramethylbensidine (TMB, Neogen Life Sciences) was added to each well, incubated for 5 minutes then stopped using NaSO_4_. The reaction was read using a microplate reader (Multiskan FC, Fisher or SynergyH1 BioTek) for absorbance at 450nm. Included on each plate were positive controls consisting of a serum from a pool of subjects that were clinically ill with COVID and tested by rtPCR positive for the virus at least 4 weeks before collection of serum as well as a human anti-SARS-CoV-2 monoclonal antibody. A negative control consisted of a pool of serum from patients from a pre-pandemic period. Two blank wells were also included in each plate and consisted of all reagents except for primary antibody.

### Statistical analysis

We estimated that with 50 samples collected before the pandemic and 50 after, we had 80% power to detect a difference in seroprevalence assuming 15% of babies would be seropositive after the beginning of the pandemic and 0% before (with 95% confidence). The average of optical densities (ODs) across the two duplicates run for each sample was calculated. We fit regression models to average ODs to determine factors associated with larger OD values including date of sample collection (represented as either year of collection or a spline term on date), age of child at sample collection, age of mother, parity of mother, educational status of mother and marital status. A mixture model was fit to the average OD’s across all samples, assuming that observations came from two distributions, those that had not been infected and those that had in the past. A threshold in OD value was identified from the mixture model that indicated a 95% probability of belonging to the distribution associated with higher values (OD>0·21). This threshold was used to define individual measurements as indicating seropositivity. Interval censored survival analysis was used to investigate the association of the hazard of SARS-CoV-2 seropositivity with date of sample collection, age of child at sample collection, age of mother, parity of child, educational status of mother. Kaplan Meier plots and piece constant hazard survival models were estimated.

## Results

### Infant cohort

In this study, 388 samples were obtained from 257 children at one or more time points including birth, approximately 4 months of age (between 4 to 8 months), approximately 12 months of age (between 8 and 18 months) and between 18 and 30 months of age from February 2019 to March 2021 (Table 1). The majority of the samples were collected at ~4 and ~12 months of age. Eighty-five samples were obtained in 2019, 199 in 2020, and 104 in 2021. Longitudinal samples were obtained from 107 of these children consisting of two or more samples: this includes 84 infants for whom two samples were available, 22 for whom three samples were available, and one for whom samples were available for all four time points.

**Table 1:** Age of infants and year of sampling of children tested for antibodies to SARS-CoV-2 sampled in the Gessier region between June 2019 and March 2021.

Year	Birth	4–8 months	8–18 months	18–30 months	Total
2019	14	54	17	0	85
2020	9	66	117	7	199
2021	0	0	56	48	104
Total	23	120	190	55	388

^1^Infants were enrolled to be sampled at birth, 4 months, 12 months and 24 months, however sampling variation occurred due to local political, climatic, and geological events in Haiti over the course of the study.

### Protein measurements

Because the blood spots can vary in size and intensity depending on difficulties encountered with collection in children, we wanted to confirm that there was detectable protein in each sample. The average protein concentration for all samples was 5,194 μg/mL with 18 measuring below 100 ug/mL and the highest measuring 13,209 μg/mL. There were no differences in mean protein levels when analyzed for age or year.

### Infant data

Using the dataset of all samples (388), a spline investigating the relationship between time of sampling and OD indicated that average ODs increased ~0·200 in value at 400 days past January 1, 2019 or approximately March 2020 (S1 Fig). Increases in ODs that occurred in 2020 and 2021 reflected the timing of waves of reported SARS-CoV-2 cases and deaths in Haiti over the same time periods (Fig 1). No anti-SARS-CoV-2 IgG responses above the predicted cut-off occurred before July, 2020 and no infant was seropositive at birth. Forty-three (16·7% [95% CI 12·7%-21·8%]) of 257 unique children tested seropositive at some point during the study.

**Fig 2:**
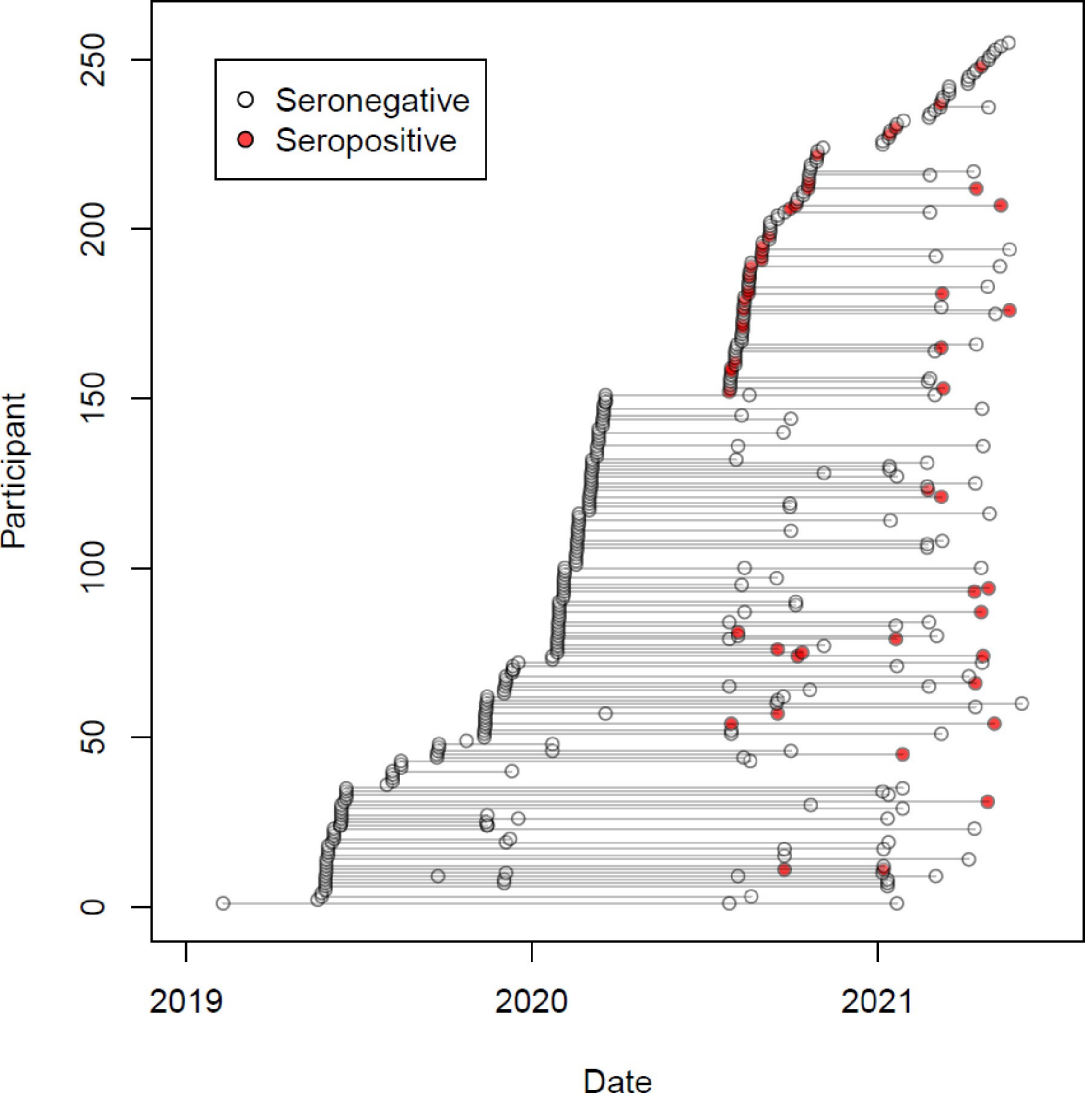
Times of follow up for each infant with serostatus indicated from 2019 to 2021. Seropositive samples are shown in red and the seronegative are without fill. Lines connect samples from the same infant.

**Fig 3:**
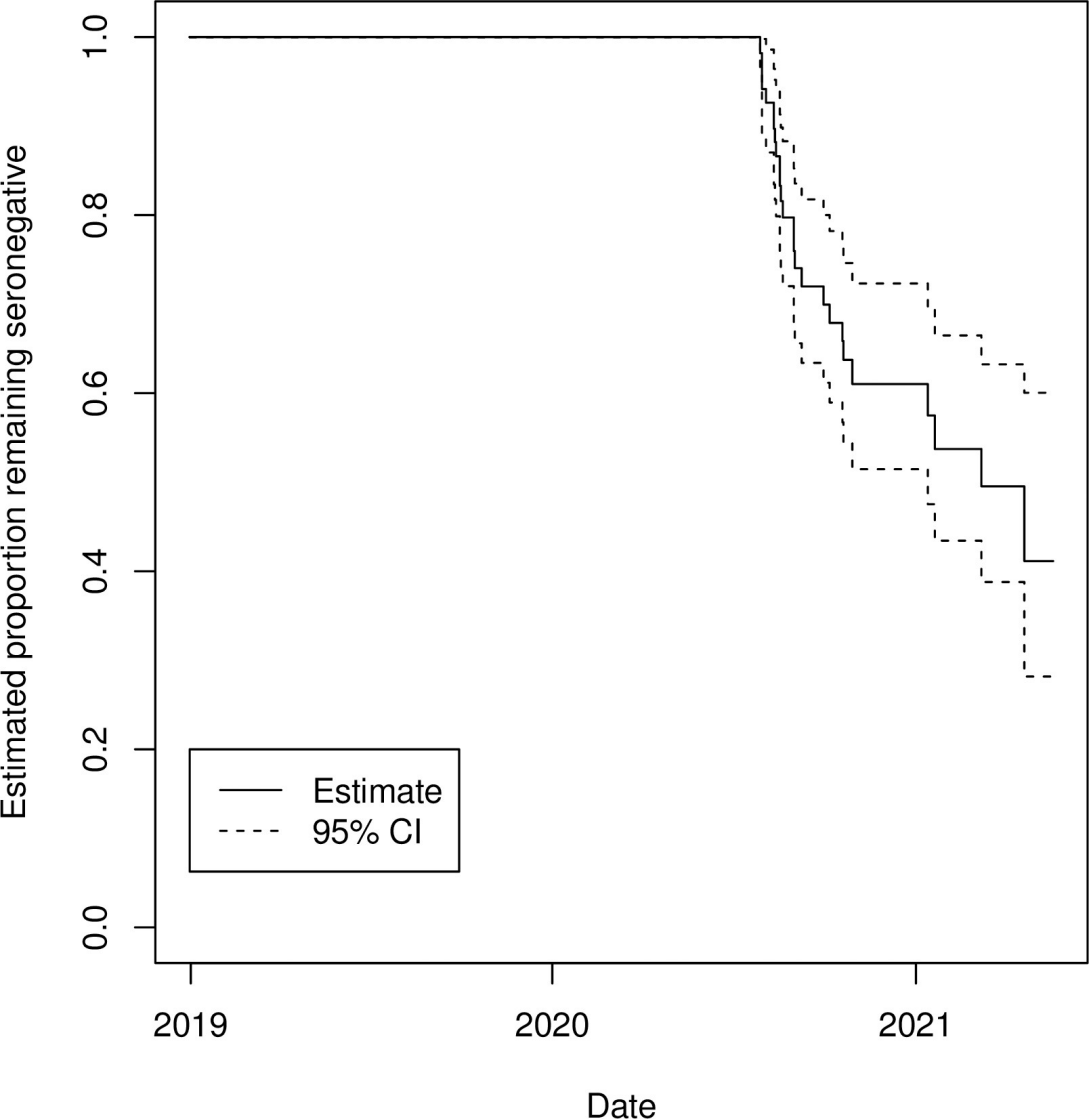
Kaplan Meier survival plot showing estimated probabilities of individuals in our cohort remaining seronegative. Estimates account for interval censoring in data.

We conducted a survival analysis to account for differing amounts of person time for each individual and over time (Figs 2 and 3). Fig 3 shows a Kaplan Meier plot indicating the proportion of individuals remaining seronegative at different times in the study. We fit piecewise constant hazard survival models to estimate the hazard of anti-SARS-CoV-2 seropositivity over time in our study population. Hazards estimated for intervals before and after March 1, 2020 found that hazards were significantly larger in the later interval (Hazard ratio of 39·1 95% CI 13·1, 2.8e7). Age of child, mother’s age, educational attainment, marital status, urban/rural status and parity of birth were not associated with the hazard of infection. Among samples taken after the first case of COVID-19 was reported in Haiti (March 19, 2020), 23% were seropositive. Trends in OD were similar across age groups (S2 Fig).

Forty-seven of the consecutively sampled children had their first sample in 2019 with the remainder enrolled and sampled after March 2020. Twenty-one of the consecutively tested children tested above the cut-off in 2020 and 2021, with seven testing above the cut-off on two of the samples. Only one child (of 8 total) who had a sample collected after an initial seropositive sample became seronegative upon follow up.

Only limited data were available on clinical illnesses experienced by children during the course of the study. However, no child was diagnosed at a medical facility as having COVID-19, based either on clinical presentation or laboratory testing, and mothers did not spontaneously report occurrence of more severe illnesses in their infants that might have been consistent with COVID-19.

## Discussion

To our knowledge this is one of the first seroprevalence studies composed of non-hospitalized children less than two years of age in a LMIC in which longitudinal sampling was conducted before and after the onset of the pandemic. Sampling date was the only covariate associated with the hazard of seroconversion, with all positive samples occurring after the start of the COVID-19 pandemic in March, 2020. We looked at a range of other maternal and child risk factors, and found none that correlated with seropositivity or seroconversion: specifically, risk of a positive serologic response did not correlate with age of the infant, or maternal characteristics such as marital status, parity, educational level, or residence in urban vs. rural settings (although most of our participants were from rural settings, and “urban” settings did not include Port-au-Prince, but, rather, small towns in the Gressier region). The overall seropositivity rate of 16.7% was lower than the 39% seroprevalence reported by our group for adult populations in Haiti during a comparable time period [11]. This may be a reflection of lower exposure of neonates and very young children to the general population. Also, the adult data in the previous publication were collected from predominantly urban populations in Port-au-Prince, including patients recruited from medical clinics. Given that studies in Peru and South Africa have demonstrated higher seroprevalence rates in more densely populated urban areas as compared with rural areas [5, 6], caution should be used in directly comparing the data from our infants (the majority of whom came from a rural origin) with these adult data.

In work performed in middle and upper income countries, SARS-CoV-2 seropositivity in children generally identified as below the age of five has been highly variable, from less than 1% in German and Switzerland to 6% in Spain after during the first surge of the pandemic [13–15]. Several U.S. studies examined antibody prevalence in residual blood samples of children during the late spring and early fall of 2020, with seroprevalence rates of 9·5–16·3% [16–18]. Recently, a cross sectional investigation of SARS in Virginia reported 8·5% seropositivity in a study of 1,038 children; seroprevalence was highest (13·7%) in children in the 0–5 year age group [19]. Our data are consistent with the observation that rates of seropositivity are higher in LMIC settings and underscore the widespread distribution of infection in very young children in these areas, with no risk factors identified other than occurrence in the midst of an epidemic wave.

In keeping with data showing little evidence of serious clinical illness in infected infants [20], we did not obtain a history of a serious preceding illness among our seropositive infants, nor were any of the children in the study formally diagnosed as having COVID-19. However, our data document that infections do occur in infants and elicit an immune response. These studies were conducted at a time when B.1 lineage strains were predominant in Haiti and need to be repeated with the successive waves of Gamma, Delta, and Omicron strains which have occurred [11]; the health impact of these later lineages also remains to be determined. However, given that infants have been shown to have high SARS-CoV-2 viral loads [9], our findings underscore the potential importance of very young children in facilitating virus transmission within LMIC households and communities, and the need to consider these youngest members of society in developing models and prevention strategies for COVID-19.

## Supporting information

S1 Fig Density of observed OD values with distributions identified by a mixture model that assumed the values were drawn from two distributions, one that we associated with negative response (red) and positive response (green). (PDF)

S2 Fig Times of follow up for each infant with serostatus indicated from 2019 to 2021. Seropositive samples are shown in red and the seronegative are without fill. Shape of points indicate age of participant (circles, at birth, squares ~4 months, diamonds ~12 months, triangles ~24 months). Lines connect samples from the same infant.
(PDF)

S1 File Inclusivity in global research. (DOCX)
