# Supplementary figures and images for: SARS-CoV-2 infections in infants in Haiti 2020–2021; evidence from a seroepidemiological cohort
Source: PLoS One. 2022 Aug 25;17(8):e0273482. doi: 10.1371/journal.pone.0273482 (PMC9409576; doi:10.1371/journal.pone.0273482)

## Density Curves

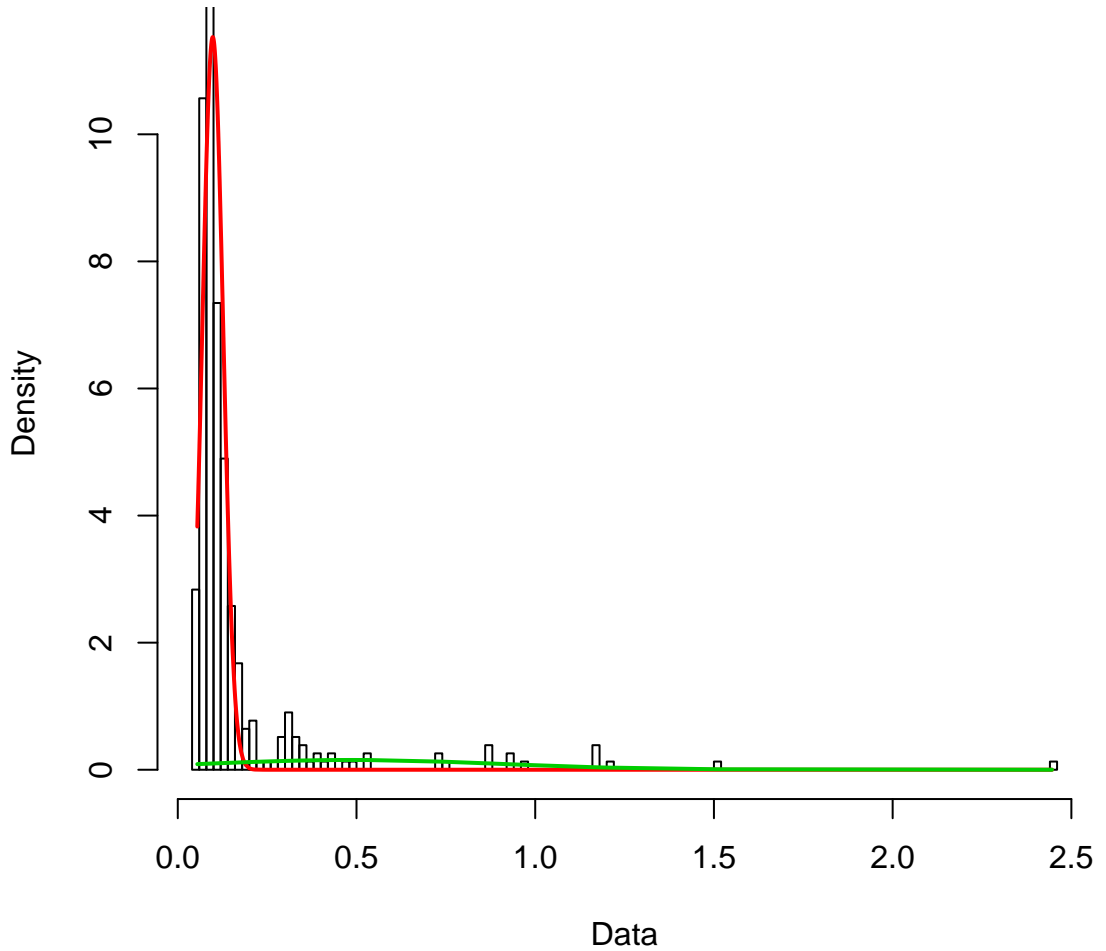

Supplement: S1 Fig — (PDF) [file pone.0273482.s001.pdf]

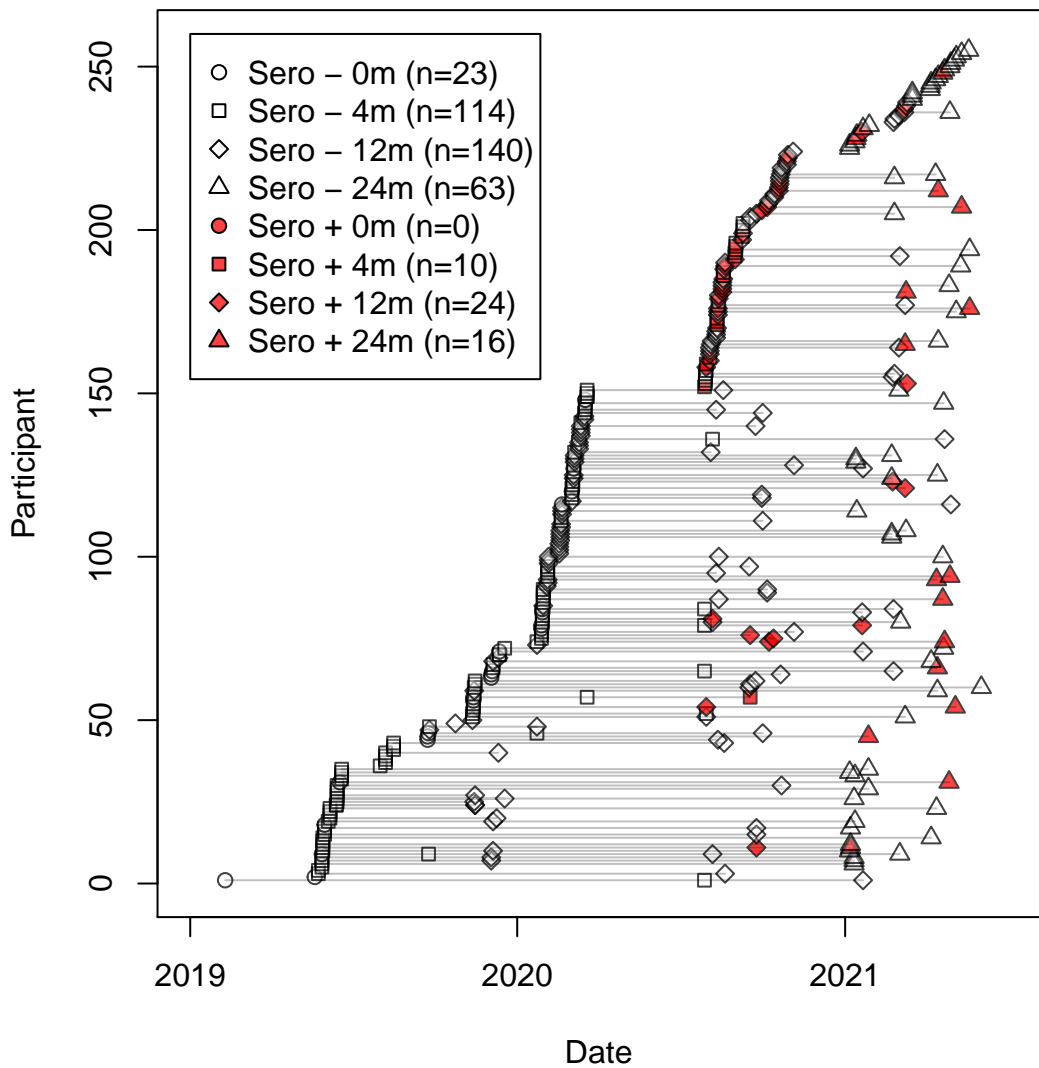

Supplement: S2 Fig — Seropositive samples are shown in red and the seronegative are without fill. Shape of points indicate age of participant (circles, at birth, squares ~4 months, diamonds ~12 months, triangles ~24 months). Lines connect samples from the same infant. (PDF) [file pone.0273482.s002.pdf]
